# The lytic transglycosylase, LtgG, controls cell morphology and virulence in *Burkholderia pseudomallei*

**DOI:** 10.1038/s41598-019-47483-z

**Published:** 2019-07-30

**Authors:** Christopher H. Jenkins, Russell Wallis, Natalie Allcock, Kay B. Barnes, Mark I. Richards, Joss M. Auty, Edouard E. Galyov, Sarah V. Harding, Galina V. Mukamolova

**Affiliations:** 1 Department of Infection, Immunity and Inflammation, University of Leicester, Leicester, UK; 2 Defence Science and Technology Laboratory, Chemical, Biological and Radiological Division, Porton Down, Salisbury, Wiltshire UK; 3 The Leicester Institute of Structural and Chemical Biology, Henry Wellcome Building, University of Leicester, Leicester, UK; 4 Electron Microscopy Facility, Core Biotechnology Services, University of Leicester, Leicester, UK; 5 Department of Respiratory Sciences, University of Leicester, Leicester, UK; 6 Department of Genetics and Genome Biology, University of Leicester, Leicester, UK

**Keywords:** Enzymes, Bacterial physiology

## Abstract

*Burkholderia pseudomallei* is the causative agent of the tropical disease melioidosis. Its genome encodes an arsenal of virulence factors that allow it, when required, to switch from a soil dwelling bacterium to a deadly intracellular pathogen. With a high intrinsic resistance to antibiotics and the ability to overcome challenges from the host immune system, there is an increasing requirement for new antibiotics and a greater understanding into the molecular mechanisms of *B*. *pseudomallei* virulence and dormancy. The peptidoglycan remodeling enzymes, lytic transglycosylases (Ltgs) are potential targets for such new antibiotics. Ltgs cleave the glycosidic bonds within bacterial peptidoglycan allowing for the insertion of peptidoglycan precursors during cell growth and division, and cell membrane spanning structures such as flagella and secretion systems. Using bioinformatic analysis we have identified 8 putative Ltgs in *B*. *pseudomallei* K96243. We aimed to investigate one of these Ltgs, LtgG (BPSL3046) through the generation of deletion mutants and biochemical analysis. We have shown that LtgG is a key contributor to cellular morphology, division, motility and virulence in BALB/c mice. We have determined the crystal structure of LtgG and have identified various amino acids likely to be important in peptidoglycan binding and catalytic activity. Recombinant protein assays and complementation studies using LtgG containing a site directed mutation in aspartate 343, confirmed the essentiality of this amino acid in the function of LtgG.

## Introduction

*Burkholderia pseudomallei* is a Gram-negative, rod shaped saprophytic bacterium that resides in soil and water environments of tropical and subtropical regions of the world. When introduced into the human host, typically via a dermal wound or inhalation, *B*. *pseudomallei* can result in the disease melioidosis. The severity of the disease increases in people in higher risk groups such as those with diabetes, renal disease and cystic fibrosis^1,2^. Systemic infection can present as pneumonia, bone and joint aches and soft tissue infections, later followed by multiple-organ abscesses and, in severe cases, septicaemia and death. Current estimates, calculated using an epidemiological model, suggest that there are as many as 165,000 melioidosis cases per annum resulting in approximately 89,000 fatalities^3^. Thus, melioidosis has a very high mortality rate compared with other infectious diseases.

The treatment of melioidosis is difficult due, in part, to the intrinsic resistance of *B*. *pseudomallei* to many antibiotics including penicillins, cephalosporins, rifamycins, and aminoglycosides^4^. The intracellular nature of *B*. *pseudomallei* also poses challenges for treatment. Both of these factors result in long courses of combinational antibiotic treatment. Eradication of the bacteria from the body is essential to prevent the development of latent infection.

The ability of *B*. *pseudomallei* to cause latent infection is relatively well documented and became of particular interest following the Vietnam War. A number of United States veterans began showing symptoms of the disease many years after the war, particularly helicopter crew members who are believed to have been exposed to aerated bacteria from soil and contaminated waters disturbed by rotor blades^5,6^. It is widely hypothesised that these latent infections are caused by bacteria in a special dormant state. These dormant bacteria are unable to grow in conventional laboratory media and are therefore referred as “non-culturable”^7^ or differentially culturable^8^ cells. The phenomenon of differential culturability is an emerging area in microbiology and the number of bacteria known to employ this survival mechanism is growing^9^. There is a family of proteins that have been shown to enable growth of differentially culturable mycobacteria^10–12^. These proteins, known as resuscitation-promoting factors (Rpfs), are a class of cell wall remodelling enzymes called lytic transglycosylases (Ltgs) which are ubiquitous to bacteria and some bacteriophages^13^.

Ltgs cleave the β-1,4 glycosidic bond between alternating N-Acetylmuramic acid (MurNAc) and N-Acetyl-D-glucosamine (GlcNAc) residues that make up the peptidoglycan backbone. Unlike lysozymes, which share a common substrate, Ltgs are non-hydrolytic enzymes and instead cleave the glycosidic bond to form an 1,6-anhydro-muramoyl peptide^14^. These proteins have been shown to have a number of functions in bacteria including cell wall recycling and expansion of the sacculus and the insertion of macromolecular structures such as the Type III and VI secretion systems, flagella, transport systems and type IV pili^14–16^. In addition, these proteins play a role in antimicrobial susceptibility in a range of bacterial species including *Pseudomonas aeruginosa*^17^, *Campylobacter jejuni*^18^ and *Escherichia coli*^19^.

There are 6 families of Ltgs in Gram-negative bacteria with many species encoding multiple Ltgs from several Ltg families^13,20^. In *E*. *coli*, for example, eight genes encode individual Ltgs (MltA-G and Slt70)^14,21^. Despite biochemical characterisation of these enzymes their specific biological roles remain unclear.

In this study, we used bioinformatic analysis to identify 8 putative Ltgs in *B*. *pseudomallei* strain K96243. We focused our studies on one of these proteins, LtgG (BPSL3046). Characterisation of an *ltgG* mutant revealed a significant role in cell division, a phenotype that could be enhanced through deletion of further *ltg* genes. LtgG involvement in flagella mediated motility and virulence in a BALB/c model of melioidosis was also demonstrated. Determining the X-ray crystal structure of LtgG in combination with site directed mutagenesis studies has identified the Asp343 residue as a key amino acid in the activity of the protein. Furthermore, complementation studies demonstrated that this residue in critical for function of LtgG. This is the first investigation of lytic transglycosylases in *B*. *pseudomallei* and we present evidence that the peptidoglycan cleaving activity of one of these (LtgG), influences multiple biological processes.

## Results

### Bioinformatics analysis identified multiple Ltg homologues in *B*. *pseudomallei*

**Figure 1:**
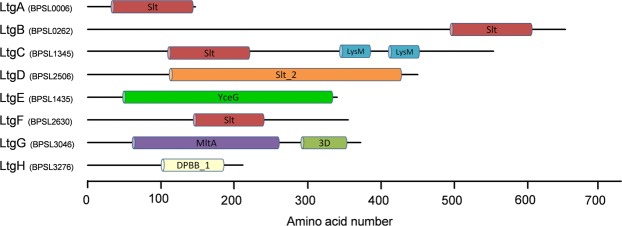
Schematic representation of predicted Ltgs in *B*. *pseudomallei* K96243. Key domains are highlighted and were identified using Pfam database and Interpro. Slt; Pfam PF01464, LysM; Pfam PF01476, Slt_2; Pfam PF13406, MltA; Pfam PF03562, 3D; Pfam PF06725, DPBB_1; Pfam PF03330, YceG; Pfam PF02618.

*B*. *pseudomallei* strain K96243 possesses at least 8 putative Ltgs (Supplementary Table S1). They are encoded by genes located across chromosome one and are not associated with the genomic islands of *B*. *pseudomallei*^22^. According to published classification^20^, these proteins belong to different families, as determined by the architecture of the Ltg domains (Fig. 1 and Supplementary Table S1). Four proteins have the SLT_1 domain and belong to family 1; LtgA (BPSL0006), LtgB (BPSL0262), LtgC (BPSL1345) and LtgF (BPSL2630). Other Ltgs identified were LtgG (BPSL3046), LtgD (BPSL2506), LtgE (BPSL1435) and LtgH (BPSL3276). LtgH shares sequence homology with RlpA of *P*. *aeruginosa*, which has a conserved catalytic aspartate and is likely to possess enzymatic activity^13^. Many of these proteins are highly conserved in bacteria with much focus on homologues in *E*. *coli* and *P*. *aeruginosa* in addition to Gram-positive Firmicutes^13^. All of the Ltgs identified in *B*. *pseudomallei* have predicted signal sequences for SecA-mediated secretion into the periplasm. Other features include a peptidoglycan-binding domain LysM^23^ in LtgC and a catalytic 3D domain^24^ in LtgG. The catalytic sites were predicted to be either a conserved glutamate or aspartate residue (Supplementary Table S1). The presence of multiple Ltgs may suggest functional redundancy, however the results of this study demonstrate that LtgG has specific roles in cell division, motility and virulence of *B*. *pseudomallei*. The important role of LtgG in *B*. *pseudomallei* biology was emphasised through the characterisation of a multiple *ltg* deletion mutant (a full strain list can be seen in Supplementary Table S2). Structural studies and initial characterization of enzymatic activity provide further insights into the molecular mechanisms underlying the function of LtgG in *B*. *pseudomallei*.

### Deletion of *ltgG* results in the generation of elongated cells

**Figure 2:**
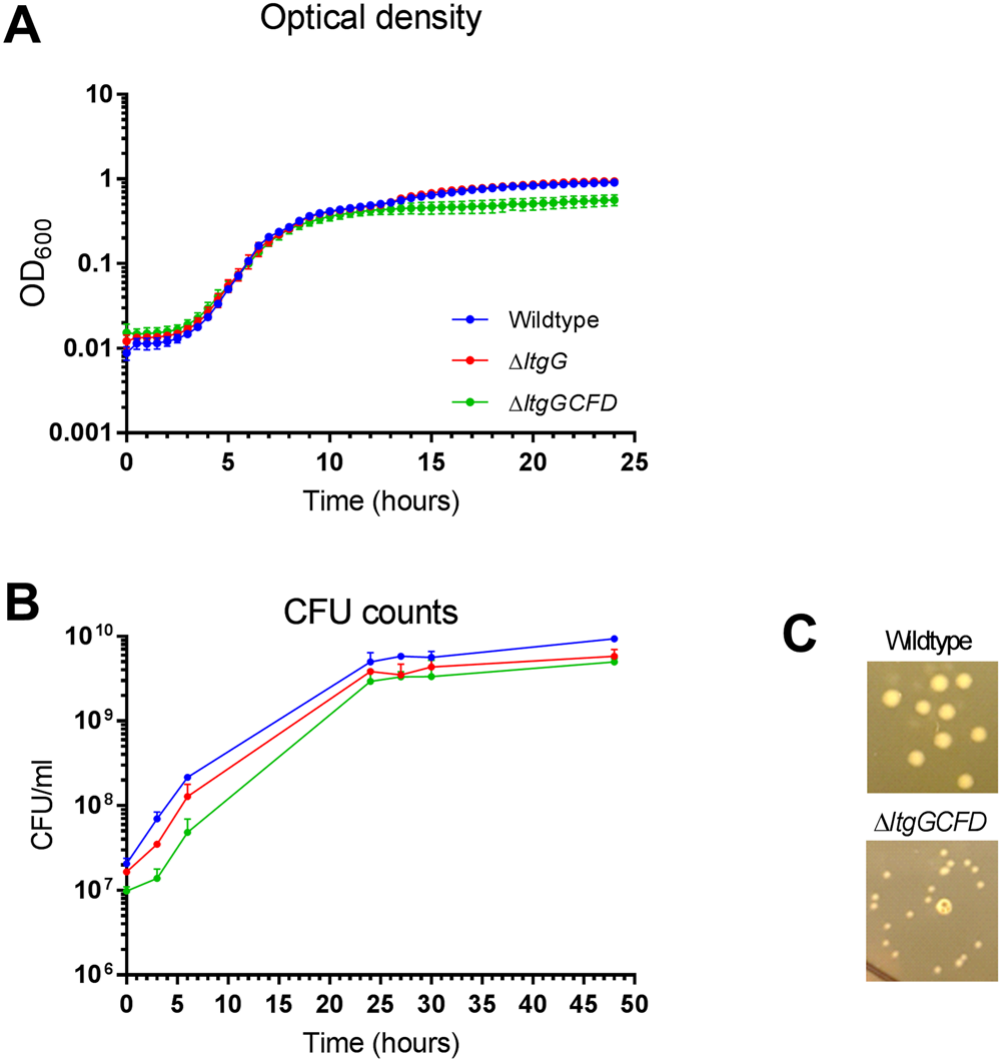
Multiple *ltg* deletion impacts on growth of *B*. *pseudomallei*. Growth of *B*. *pseudomallei* strains in LB was assessed by measuring optical density at 600 nm (**A**) or by CFU counting (**B**). (**C**) *B*. *pseudomallei* were grown on LA plates for 36 h. Data are represented as mean ± STDV (n = 7 for A, n = 2 for B). Analysis of growth parameters is presented in Supplementary Table S3.

Initial studies with an *ltgG* mutant (∆*ltgG*) in nutrient rich Lysogeny broth (LB) did not reveal a growth defect (Fig. 2). The calculated growth rate was 0.591 ± 0.026 h^−1^ compared to 0.619 ± 0.054 h^−1^ for the wildtype strain (Supplementary Table S3). In contrast, a multiple *ltg* deletion mutant lacking four of the eight *ltg* genes, ∆*ltgGCFD*, revealed a small but significant growth defect determined by OD and CFU counts (Fig. 2A,B). It had a slightly impaired growth rate (0.558 ± 0.022 h^−1^). The growth rate, doubling time and OD at 24 hours for ∆*ltgGCFD* were significantly different from those of wildtype (p < 0.05). ∆*ltgGCFD* colonies were also notably smaller than those of wildtype which required 24 hours of additional incubation to grow (Fig. 2C). ∆*ltgGCFD* however had no survival defect in stationary phase (Fig. 2B).

**Figure 3:**
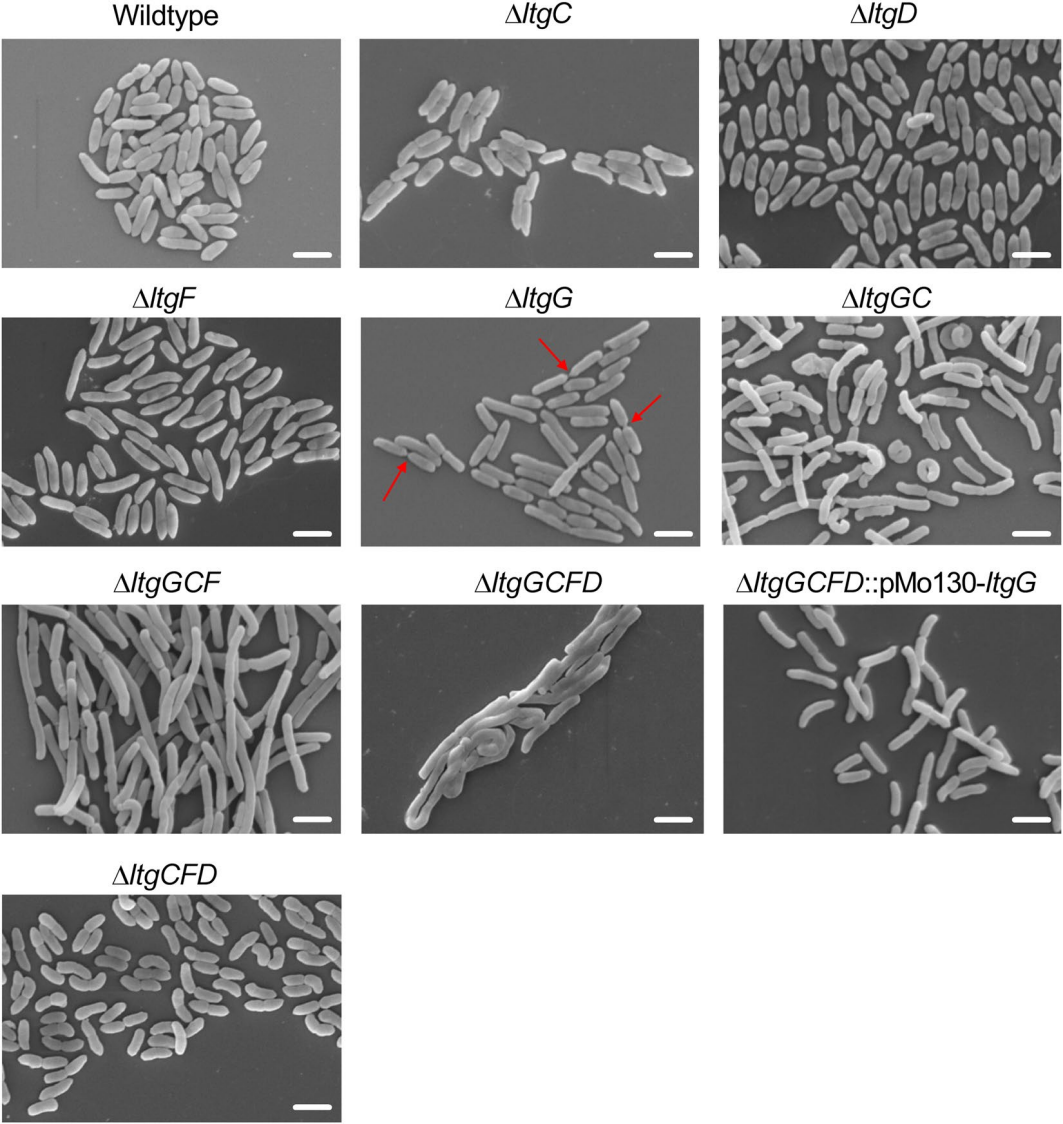
Deletion of *ltgG* results in generation of elongated cells. *B*. *pseudomallei* strains were grown to early exponential phase and fixed in glutaraldehyde as described in Methods. For assessment of morphology 10 independent fields were monitored, representative images are shown. Scale bar is 2 µm. Examples of unseparated cells are marked with arrows.

The effect of mutating *ltgG* on the morphology of *B*. *pseudomallei* was investigated using scanning electron microscopy (SEM). Δ*ltgG* demonstrated an increased cell length and the presence of unseparated cells compared to the wildtype strain, suggesting the involvement of LtgG in cell growth and division (Fig. 3 and Table 1). The consequence of this defect led to the occasional chaining of cells up to 20 bacteria in length. The elongated ∆*ltgG* cells were more prominent upon subsequent deletion of additional *ltg* genes, resulting in vast filament-like cells comprising of chains of bacteria, varying in length. There were no differences in cellular morphology (cell length or unseparated cells) for any of the single *ltg* mutants.

**Table 1:** Extended Cell Morphology is due to the *LtgG* Mutation.

Strain	Mean cell length ± SEM^a^	Minimum/maximum (µm)
Wildtype	2.3 (0.6)	1.0/5.1
∆*ltgG*	3.1 (1.1)	1.5/12.7
∆*ltgGC*	4.3 (1.9)	1.5/21.9
∆*ltgGCF*	7.1 (3.6)	1.4/36.6
∆*ltgGCFD*	7.98 (4.9)	2.02/45.44
∆*ltgG*::pMo130-*ltgG*	2.21 (0.78)	0.87/7.72
∆*ltgGCFD*::pMo130-*ltgG*	2.89 (1.38)	0.92/15.27
∆*ltgCFD*	2.68 (0.8)	1.25/8.52

^a^Bacteria were visualised by phase microscopy and the cell length measured for all bacteria in 20 different fields of view.

Phase contrast microscopy was used to quantify the differences in the cell length of ∆*ltgG* and the multiple *ltg* mutant. The mean cell lengths and standard errors are detailed in Table 1. Deletion of *ltgG* resulted in a 50% increase in mean cell length compared to wildtype, consistent with the morphological differences demonstrated by SEM. Further deletions caused an additive effect with ∆*ltgGC* cells being 85% longer in length than wildtype and ∆*ltgGCF* cells 300% longer. The quadruple deletion, ∆*ltgGCFD*, led to an average cell length 346% longer than wildtype.

Given that ∆*ltgG* was the only single deletion mutant that demonstrated differences in cell morphology, this gene was reintroduced, *in cis*, to ∆*ltgGCFD* creating *∆ltgGCFD::pMo130-ltgG*. This was carried out to determine the contribution of *ltgG* to the formation of these filament-like cells. Comparison of ∆*ltgGCFD::*pMo130-*ltgG* to ∆*ltgCFD* shows that the bacteria were of a similar length; 2.89 and 2.68 µm, respectively. Furthermore, wildtype and ∆*ltgCFD* were of similar lengths, 2.3 µm and 2.68 µm respectively. Together, these data suggest that LtgG has a major role in maintaining the cell length and morphology of *B*. *pseudomallei* and contributes to bacterial growth.

### *∆ltgG* has defects in swimming and swarming motility but not in biofilm formation and antibiotic susceptibility

**Figure 4:**
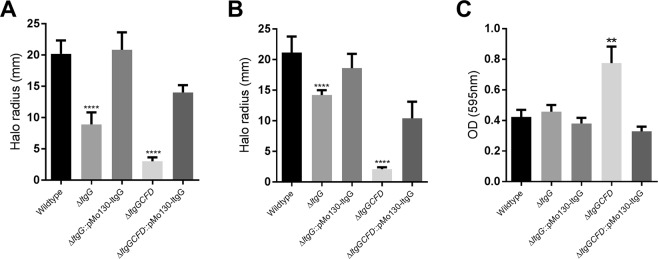
Δlt*gG* has significantly reduced swimming and swarming motility but not biofilm production. Swimming (**A**) and swarming (**B**) motility assays were performed as described in Methods. For assessment of biofilm formation bacteria were stained with crystal violet and absorbance of ethanol extracts measured at 595 nm (**C**). Motility data are shown as mean values with error bars representing standard deviation, three biological replicates with 3 technical replicates per experiment were performed. Biofilm data are shown as mean values with error bars representing standard error, four biological replicates with 8 technical replicates were performed per experiment. One-way ANOVA analysis was performed comparing mutant strains to wildtype, **p < 0.01, ****p < 0.0001.

We next investigated whether *ltgG* deletion also affected other important features including motility and biofilm formation of *B*. *pseudomallei*. Results of experiments detailed in Fig. 4 demonstrated that Δ*ltgG* displayed significantly reduced swimming and swarming motility (p < 0.0001) with a 56% and 33% reduction from wildtype, respectively. As in the experiments described above, *in cis* complementation with *ltgG* restored the wildtype phenotype. Interestingly, ∆*ltgGCFD* was non-motile but motility was partially restored through complementation with *ltgG*. Furthermore, the quadruple *ltg* mutant demonstrated increased biofilm production which was reduced to wildtype levels by complementation with *ltgG* (Fig. 4C). Δ*ltgG*, however, was not affected in its ability to produce biofilms and levels remained similar to that of wildtype bacteria (Fig. 4C). In addition, the inactivation of multiple *ltgs* resulted in a very modest two-fold increase of susceptibility to carbenicillin and ceftazidime (Table 2).

**Table 2:** Minimum inhibitory concentrations of Ltg mutants of *B. pseudomallei* for selected antimicrobial agents.

	WT	ΔltgC	ΔltgD	ΔltgF	ΔltgG	ΔltgGCFD	∆ltgGCFD:: pMo130-ltgG
Meropenem	2	2	2	2	2	2	2
Imipenem	1	1	1	1	1	1	1
Co-trimoxazole	64	64	64	64	64	64	64
Carbenicillin	1024	1024	1024	1024	1024	512	512
Ceftazidime	16	16	16	16	16	8	8
Doxycycline	2	2	2	2	2	1	1

Antibiotic susceptibility was measured by determining the MIC by serial dilution method. Results are from triplicate experiments (four replicate wells per experiment). Concentrations are in µg/ml. WT – wildtype strain.

### The ltgG mutant is attenuated a BALB/c mouse model of melioidosis

**Figure 5:**
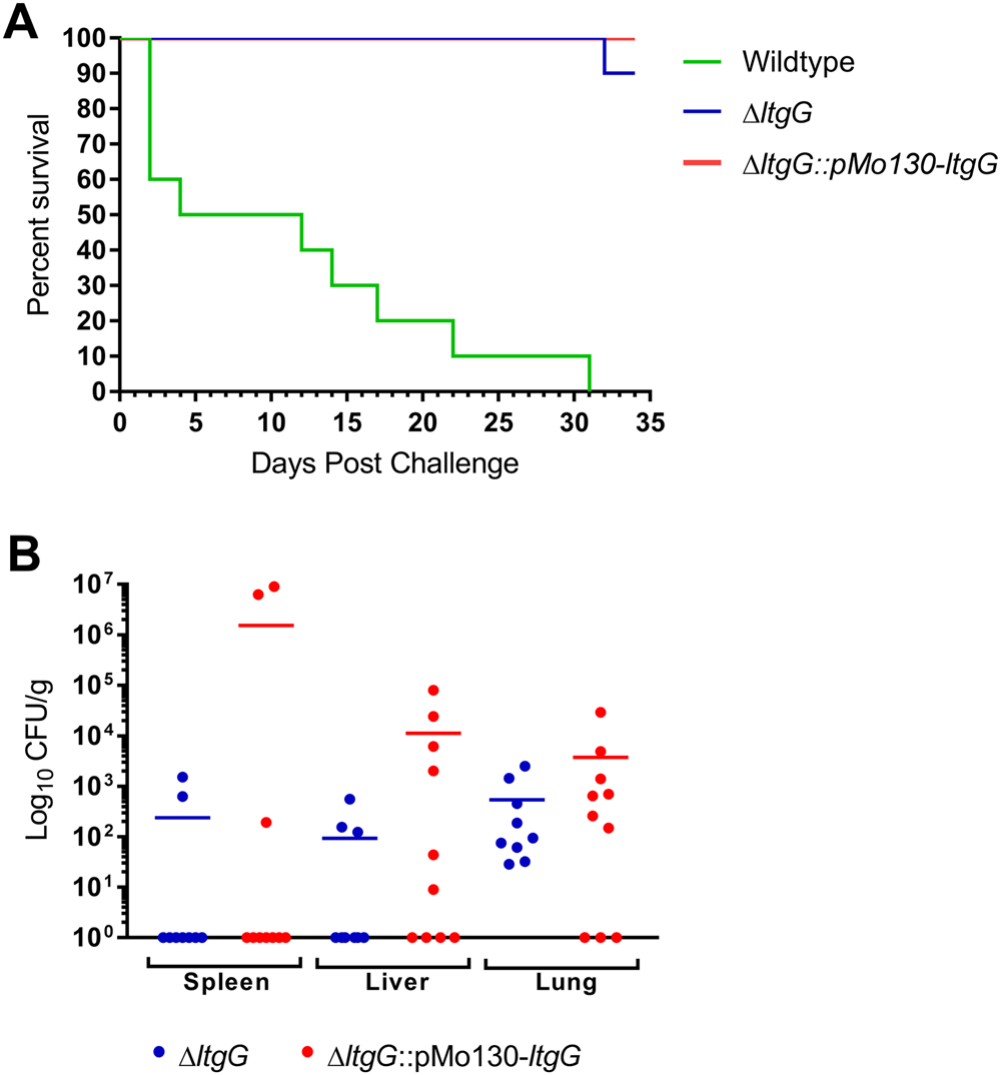
*B*. *pseudomallei* ∆*ltgG* is significantly attenuated in the IP BALB/c mouse model of infection. (**A**) Mice challenged with approx. 2 × 10^4^ CFU were monitored over 35 days. Mice challenged with ∆*ltgG* had a 90% survival. Mantel-Cox Log Rank test value of 0.001. (**B**) Organ counts from surviving mice revealed low levels of bacteria primarily in the lungs.

To evaluate the role of LtgG in virulence of *B*. *pseudomallei*, BALB/c mice were infected by the intraperitoneal route with wildtype, ∆*ltgG* or ∆*ltgG*::pMo130-*ltgG* (challenge doses; wildtype – 2.0 × 10^4^, ∆*ltgG* – 1.9 × 10^4^, ∆*ltgG*::pMo130-*ltgG* – 1.4 × 10^4^ CFU). By the end of the study (day 35), all mice infected with the wildtype strain had succumbed to infection and ∆*ltgG* was significantly attenuated with 90% survival of infected animals (Fig. 5A). Complementation of the *ltgG* mutation failed to restore virulence in this mouse model. At the end of the study, the spleen, liver and lungs of the surviving mice were harvested and the bacterial loads were enumerated. There was no statistically significant difference between the ∆*ltgG* and ∆*ltG*::pMo130-*ltgG* bacterial loads in any of the tested organs. Reduced bacterial counts were detected in the spleens, confirming that both strains were unable to colonise the spleens in the majority of the mice, while substantial numbers of bacteria were recovered from the infected lungs (Fig. 5B).

### LtgG possesses muralytic activity

We next confirmed that recombinant LtgG had peptidoglycan cleaving activity using two approaches. Firstly, LtgG was able to digest lyophilised *Micrococcus luteus* which was demonstrated by the production of a clearance band in zymography (Supplementary Fig. 1). The optimal buffer for this assay was found to be 25 mM Tris-HCl pH 7, 1 mM manganese (Supplementary Fig. 1A). Secondly, muralytic activity could be demonstrated in solution in a turbidity assay. Addition of recombinant LtgG (20 µg/ml) to a suspension of *M*. *luteus* resulted in a decrease in OD (Fig. S1B), suggesting digestion of lyophilised *M*. *luteus*. LtgG could be inactivated by heating at 95 °C. Given the shared substrate of Ltgs and lysozyme, a lysozyme positive control was included which demonstrated a very rapid decrease in OD. LtgG was able to cleave FITC-labelled peptidoglycan from *E*. *coli* (Supplementary Fig. 1C), however no soluble muropeptides were detected using HPLC.

### The structure of LtgG predicts aspartate 343 as a catalytic residue

**Figure 6:**
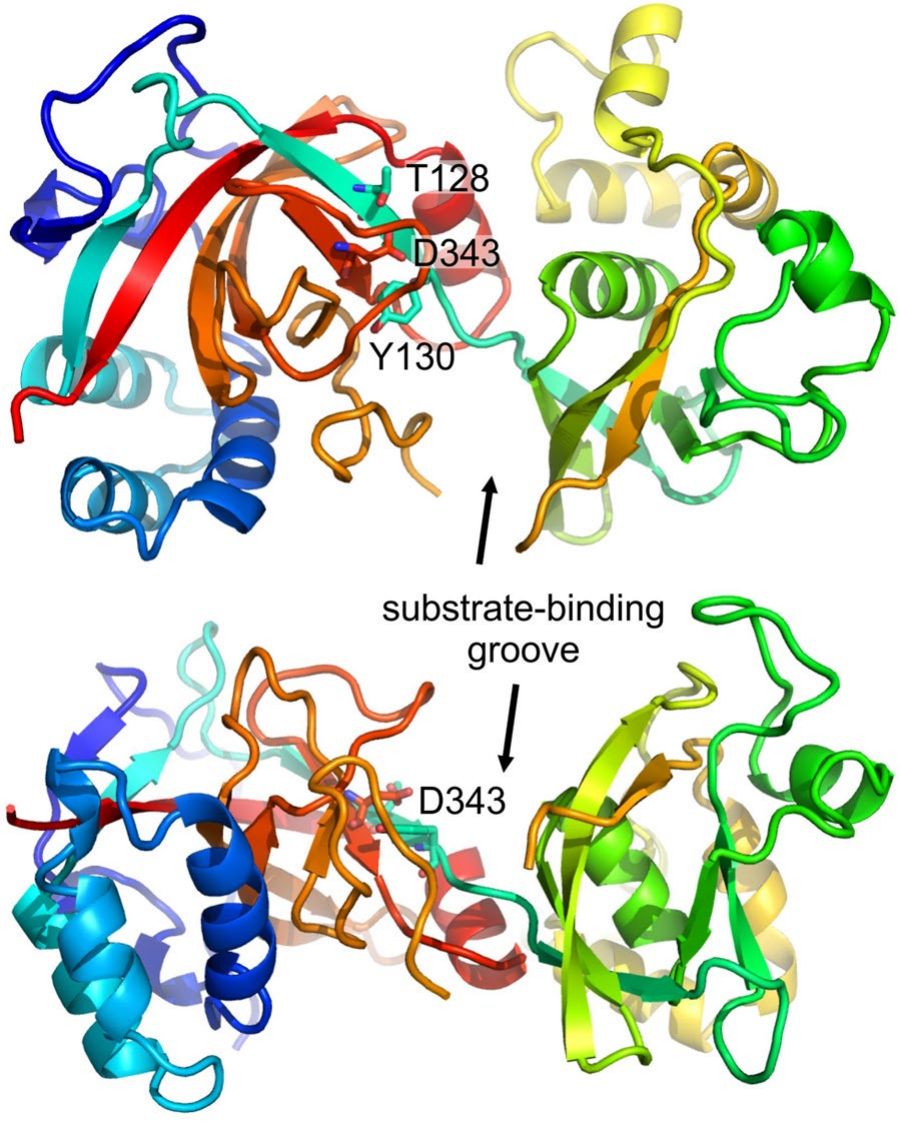
Crystal structure of LtgG at 1.7 Å resolution. Top and side views of LtgG showing the likely substrate binding groove and the essential aspartate residue, D343. Conserved residues proposed to form part of the catalytic site in the *E*. *coli* protein, MltA, are indicated.

Crystals of LtgG were grown at pH 7.5 and diffracted to 1.73 Å resolution. LtgG is comprised of two domains, a large β-barrel domain and a smaller α-/β-domain that is an insert of the larger domain (Fig. 6). The arrangement of the domains creates a wide groove, similar to that of the MltA structures of *E*. *coli* and *N*. *gonorrhoeae* (PDB: 2GAE and 2G6G), which likely contains the substrate binding and catalytic sites. Residues proposed to form part of the catalytic site in the *E*. *coli* protein, including the catalytic aspartate (Asp343 in LtgG), together with threonine (Thr128) and tyrosine (Tyr130) residues are conserved in LtgG, suggesting a common catalytic mechanism. Full collection data and refinement statistics can be seen in Supplementary Table S4.

A notable difference between the structures of LtgG and its *E*. *coli* and *N*. *gonorrheoeae* homologues is the relative orientations of the two domains, with rotations of ~40° and 20° respectively and in opposite directions (Supplementary Fig. 2A) Moreover, comparison of the LtgG with a structure of *E*. *coli* MltA bound to a chitohexaose substrate^25^ suggests that large conformational changes are likely to occur upon substrate binding. Most notably, the binding groove is narrower in the substrate bound structure, allowing interactions with both domains. (Supplementary Fig. 2B).

### The D343A mutant is not active in a zymogram and does not complement the *∆ltgG* phenotype

**Figure 7:**
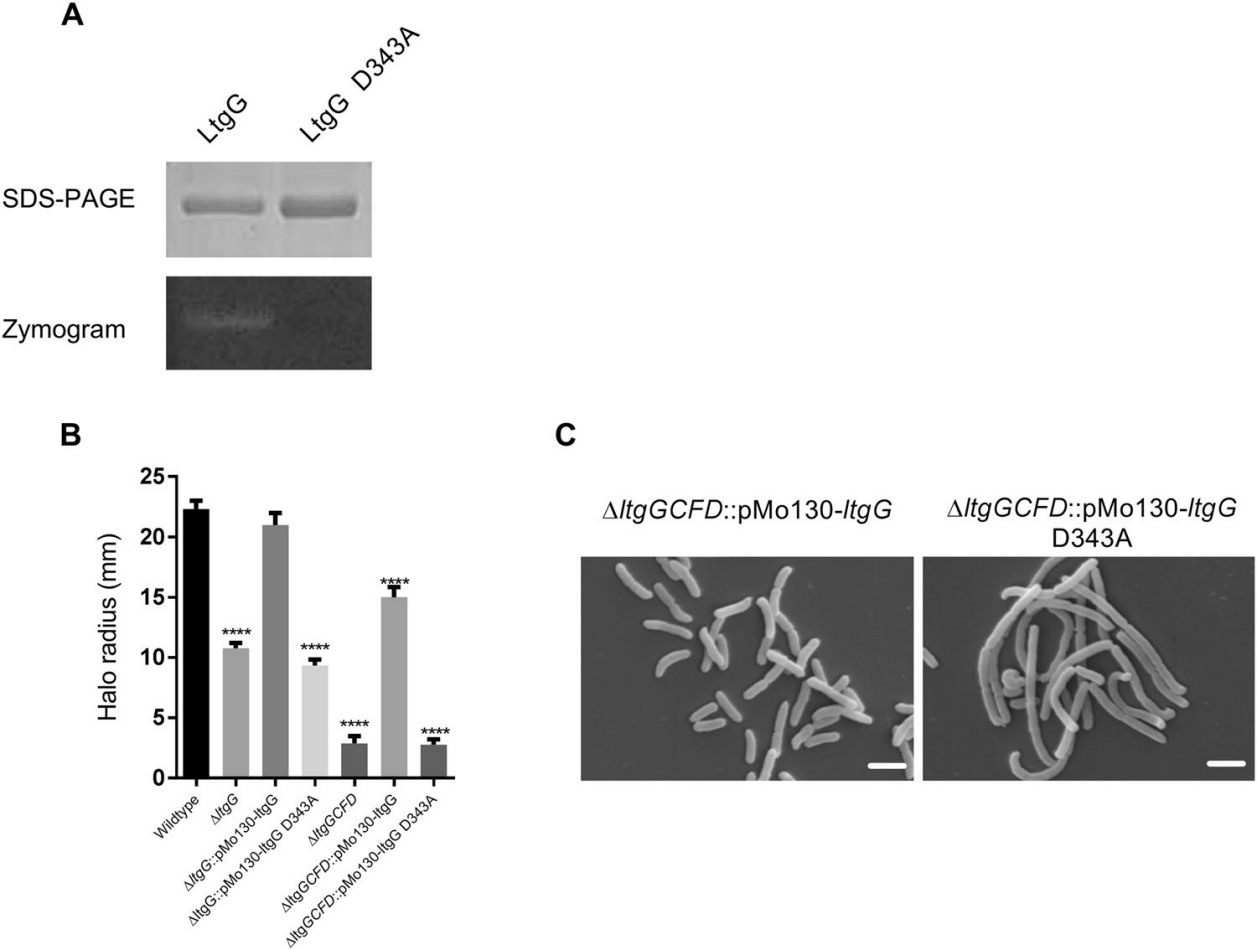
D343A mutation in LtgG abolished its activity and failed to complement mutant’s phenotype. (**A**) Zymography of wildtype LtgG and D343A variant (see Fig. S3 for uncropped images). (**B**) Complementation of swimming motility in *ltgG* mutants. (**C**) SEM micrographs of ∆*ltgGCFD* and complemented mutants. Scale bar is 2 µm. A one-way ANOVA was performed comparing LtgG mutant strains with wildtype (n = 7), ****p < 0.0001.

To confirm that the aspartate at position 343 was catalytically important, we generated a Asp343Ala (D343A) mutant protein. The mutant eluted from the gel-filtration column at the same position as the wild-type enzyme indicating that it was folded correctly. Zymograms performed using equivalent concentrations of the wildtype and mutant proteins reveal a complete loss of muralytic activity as a result of the mutation, indicated by an absence of clearance under established conditions appropriate for wildtype LtgG (Fig. 7A).

To prove that muralytic activity was important for the Ltg-mediated phenotypes we performed complementation experiments. As shown in Fig. 7B and Supplementary Fig. 3 ∆*ltgG::*pMo130-*ltgG* D343A was as motile as ∆*ltgG*. The D343A mutation completely abolished the complementation of cellular morphology in ∆*ltgGCFD*. Importantly, *ltgG* D343A was expressed at similar levels to wildtype as confirmed by RT-PCR (Supplementary Fig. 4).

## Discussion

Peptidoglycan is an integral part of a bacterial cell which provides cell integrity, protection from various exogenous stresses and accommodates many secretion systems, including those involved in virulence. Lytic transglycosylases are space-making autolysins that are essential for peptidoglycan synthesis and remodeling^14^. Ltgs are present in all peptidoglycan-containing bacteria as multiple homologues and are generally considered to be redundant for growth. Why there is a need for such a high level of redundancy and how they are regulated is so far unclear. Published studies also indicate however, that individual Ltgs may play very specific roles in various bacteria.

In *N*. *gonorrhoeae* an *ltgC* (the gene encoding a MltA/LtgG homologue) mutant displayed incomplete separation of cells during division and exhibited abnormal growth patterns compared to wildtype^26^, while LtgA and LtgD have been demonstrated to play distinct roles in muropeptide recycling and peptidoglycan remodeling^27^. Similarly, in *Staphylococcus aureus*, inactivation of *isaA*, encoding a putative Ltg, led to impaired cell separation and clumping of bacterial cultures with SceD, another putative Ltg, essential for nasal colonization of rats^28^. YocH, an Ltg in *Bacillus subtilis*, was important for survival in stationary phase^29,30^, whilst SleC Ltg was crucial for spore germination in *Clostridium difficile*^31^. Interestingly, deletion of *rpfB*, (a putative Ltg in *M*. *tuberculosis*), resulted in delayed reactivation of chronic tuberculosis in mice^32^, further supporting the importance of Ltgs for reactivation of dormant forms. Bacteria also produce highly specialized Ltgs to facilitate assembly of secretion systems and flagella^16,33,34^. Multiple *ltg* deletions usually lead to more pronounced phenotypes and can affect susceptibility to antibiotics, pathogenicity and bacterial division^17,19,28,35^. The wide range of functions highlights the importance of these enzymes for fundamental biological processes.

This study was focused on *B*. *pseudomallei*, a significant pathogen and a potential biothreat agent. Despite comprehensive characterisation of peptidoglycan-synthesising and remodeling machinery in Gram-negative bacteria^36^, very little is known about Ltgs and other cell wall enzymes in *B*. *pseudomallei*. We identified several Ltg homologues belonging to three distinct Ltg classes. Our studies have focused on four out of eight of these genes and here we have presented data primarily on LtgG as the ∆*ltgG* mutant displayed clear phenotypic differences compared with the other mutant strains.

Our results demonstrate that LtgG plays a major role in bacterial growth, cellular morphology and motility. Deletion of additional *ltgs* made these phenotypic changes even more prominent. This is not surprising since peptidoglycan is the essential component of bacteria and perturbation in its remodeling influences major biological processes^13^. In particular, ∆*ltgGCFD* had a slower growth rate in liquid media and produced smaller colonies on agar. This change in growth rate was accompanied by the accumulation of long chains of filament-like cells potentially explaining the lower number of cells and reduced OD in ∆*ltgGCFD* cultures. Interestingly, none of the mutants had a survival defect in stationary phase and showed no obvious signs of lysis. Collectively these results strongly suggest a key role of Ltgs studied here (and LtgG, in particular) in active growth, division and separation of *B*. *pseudomallei* cells. Similar morphological defects were previously observed when a single *ltgD* gene was deleted in *N*. *gonorrhoeae*^26^. This phenotype however was not seen in *E*. *coli*, in which its homologue MltA, does not play any obvious role in cellular morphology^37^. Moreover, in *P*. *aeruginosa* another Ltg, RplA (homologous to LtgH in *B*. *pseudomallei*), has been shown to be a key protein in determining both cellular morphology and daughter septation, with chaining phenotypes similar to that seen for the *ltgG* mutant^38^.

Failure to perform complete cell separation and the subsequent increase in cell length may affect multiple functions, including motility. Indeed *B*. *pseudomallei ltgG* mutant and (to a greater extent) the multiple deletion mutant Δ*ltgGCFD*, had significantly reduced motility. Furthermore, this motility defect can be caused by interference with the assembly of flagella components. Similarly, Ltgs have been shown to be required for full motility in *Helicobacter pylori*, *Salmonella typhimurium and Listeria monocytogenes*^34^ and directly shown to be involved in the assembly of flagella in *Rhodobacter sphaeroides*^39^. Remarkably phenotypic differences of ∆*ltgG* and ∆*ltgGCFD* could be restored by re-introduction of a single copy of *ltgG* into the *B*. *pseudomallei* genome.

The important role Ltgs play in virulence in a number of pathogens should not be understated and there is increasing recognition of these proteins as virulence determinants. Here, we showed that LtgG plays an important role in the virulence of *B*. *pseudomallei*. ∆*ltgG* was significantly attenuated *in vivo* with 90% mice infected with this strain surviving to the end of the study when compared to wildtype infected animals which all succumbed to infection. The bacterial load in organs from surviving animals showed that the mutant was largely unable to colonise the spleen. The reduction in motility and the chaining morphology observed with this mutant could be leading contributors to this attenuation; however, further investigation would be needed to address other contributing factors (including the assembly of secretion systems and survival in *in vivo* associated stresses). The attenuation in virulence, could not be complemented. This was also the case for a multiple *rpf* deletion mutant generated in *M*. *tuberculosis*^35^. There are many possible explanations for this phenotype including that LtgG may have specifically regulated expression in a host and the reintroduction of the gene in an alternative genome location may have affected its expression *in vivo*. Alternatively, potential instability of the integrated complementing plasmid could have resulted in loss of the plasmid during replication *in vivo*.

Surprisingly, inactivation of multiple *ltgs* caused only a modest two-fold increase in sensitivity to carbenicillin and ceftazidime. *B*. *pseudomallei* is highly resistant to many antimicrobial agents^4^ and our results suggest that Ltg-mediated peptidoglycan remodelling plays a minor role in drug susceptibility, however deletion of other Ltgs encoded by *B*. *pseudomallei* may have a more substantial effect.

To elucidate the mechanisms underlying how LtgG functions in *B*. *pseudomallei*, we purified recombinant LtgG and assessed it activity *in vitro*. As expected the protein was able to cleave the cell wall from *M*. *luteus* by zymography and a turbidometry assay confirming bioinformatic findings. LtgG was active on FITC-labelled peptidoglycan of *E*. *coli* (Supplementary Fig. 1C), however soluble muropeptides could not be reliably detected using a Prontosil 120 C18 column. Poor ability of Ltgs to release soluble muropeptides has been previously reported for several bacterial species^27,38,40^. Testing of LtgG activity in combination with additional muralytic enzymes using peptidoglycan from *E*. *coli* or *B*. *pseudomallei* will be attempted in future studies.

The significant impact of the deletion of *ltgG* on *B*. *pseudomallei* biology highlighted this protein as a valid drug target. We therefore determined the structure of LtgG and identified the potential catalytic residue as aspartate 343. This finding is in accordance with data previously published on the LtgG homologues in *E*. *coli* and *N*. *gonorrhoeae*^41^. Importantly, replacement of this residue with an alanine completely abolished muralytic activity and the complementation of the ∆*ltgG* and ∆*ltgGCFD* phenotypes. Thus, our results proved that muralytic activity of LtgG was required for its function in *B*. *pseudomallei*. The 3D domain, (in which Asp343 is located), is also present in Stationary phase survival proteins (Sps) of Firmicutes^29^. These proteins, including YabE, an Sps of *B*. *subtilis*, have been found to have the same domain organisation and a similar genomic content to RpfB in which the C’ terminal catalytic Rpf domain had been replaced with an Sps domain. This Sps domain has low, but significant sequence identity with the C terminal, 3D domain of MltA^29^. The involvement of LtgG in stationary phase survival (as for Sps) or differentially culturable cells (as for Rpfs) would require further research but there is potential for Ltgs to play an important role in the ability of *B*. *pseudomallei* to cause latent infection and reactivate from it.

Ltgs have been previously identified as being promising drug targets. NAG-thiazoline has been shown to inhibit enzymatic activity of MltB and to ifluence bacterial morphology^42,43^. Bulgecin A inhibited activity of Slt70 and LtgB^44,45^ and could increase the susceptibility of *N*. *meningitidis*, *N*. *gonorrhoeae*, *E*. *coli and H*. *pylori* to β-lactam antibiotics^45–47^. Finally, 2-nitrophenylthiocyanates (NPT) compounds were shown to inhibit the muralytic activity of resuscitation-promoting factor^48^. While these chemicals did not inhibit growth of bacteria, they could be employed to inhibit the establishment of bacterial infection and prevent reactivation of latent infections^49^. Our studies suggest that targeting Ltgs in *B*. *pseudomallei* may significantly impair its growth and virulence and offer novel therapeutic approaches for the treatment of melioidosis.

## Methods

### Bacterial strains and media

All bacterial strains and plasmids are listed in Supplementary Table S2. All media were purchased from ThermoFisher Scientific or Sigma Aldrich. Unless otherwise stated, bacteria were grown at 37 °C in Lysogeny broth (LB) at 200 rpm (*E*. *coli*) or 150 rpm (*B*. *pseudomallei*) or on Lysogene agar (LA) plates. Where necessary, the media were supplemented with appropriate antibiotics at the following concentrations: kanamycin 50 μg/ml for *E*. *coli* and 800 μg/ml for *B*. *pseudomallei*; ampicillin (100 μg/ml) or polymyxin B (15 μg/ml).

### Bioinformatics analysis

Blastp searches using Ltg protein sequences from *E*. *coli* and *P*. *aeruginosa*, and the *Burkholderia* genome database^50^ were used to identify putative Ltgs in *B*. *pseudomallei* strain K96243. Protein sequence analysis and domain architecture was performed using the Pfam database https://pfam.xfam.org and InterPro https://www.ebi.ac.uk/interpro/. Signal peptides were predicted using SignalP^51^.

### Construction of *ltg* mutants

All primers used in this study can be found in Supplementary Table S5. Unmarked *ltg* deletion mutants were generated as described previously^52^. Flanking regions were amplified by PCR using *B*. *pseudomallei* K96243 genomic DNA (gDNA) as the template and relevant primers. PCR products were purified using the QIAquick PCR purification kit (Qiagen) or by Qiagen gel extraction (Qiagen) and cloned into pMo130 between *Xba*I and *Hin*dIII sites. pMo130 was a gift from Martin Voskuil (Addgene plasmid #27388). Following sequencing at GATC Biotech, the constructs were used to transform *E*. *coli* S17-1 (*λpir*) for conjugal transfer into *B*. *pseudomallei*. Conjugal transfer of the suicide vectors was performed as described previously^52^ with the following alterations; conjugation reactions were incubated on LA for 24 hours, for *B*. *pseudomallei*, kanamycin selection was increased to (800 μg/ml) and sucrose concentration was increased to 20% (w/v). Double cross-over mutants were screened by PCR using test primers, sequencing and by Southern hybridization.

### *In cis* complementation of ∆*ltgG*

The *ltgG* open reading frame in addition to 250 bp of the upstream region containing the putative promoter and ribosome binding site was PCR amplified using the LtgG Comp130_For/Rev primers. In addition, a downstream flanking region was cloned into pMo130 using primers LtgG compFR2_For/Rev for the insertion of the plasmid into the intergenic region immediately downstream of Bpsl3330. The construct was electroporated into a donor *E*. *coli* strain; S17-1(λ*pir*) which was used in conjugation with *B*. *pseudomallei* Δ*ltgG*. Single crossover strains were selected on LA supplemented with kanamycin and polymyxin B. D343A mutations were introduced into the complementing *ltgG* construct using GeneArt® Site-Directed Mutagenesis System (ThermoFisher Scientific), as per manufactures protocol. To confirm expression of *ltgG*, reverse transcriptase PCR (RT-PCR) was performed using SuperScript® II Reverse Transcriptase kit (ThermoFisher Scientific) according to manufacturer’s protocol. RT-PCR were performed using 10-fold serially diluted RNA starting with a concentration of 1 μg. *ltgG* specific primers (LtgG_GS_For/Rev) were used for PCR amplification of cDNA. 16S rRNA specific primers (16S_For/Rev) were used as positive controls. To confirm the absence of gDNA, reverse transcriptase was excluded from the negative control.

### Analysis of *B*. *pseudomallei* growth

Overnight cultures were grown as described above and sub-cultured into 5 ml of LB (1:100 inoculum). Bacteria were enumerated by CFU counts on LA at regular intervals for 48 hours. For assessment by optical density, cultures were incubated for three hours to reach exponential phase of growth (OD_600_ of approximately 0.6) before being diluted 10-fold in LB. 10 µl was inoculated into 190 µl of LB in triplicate wells of a 96 well microtiter plate. Plates were placed into a Multiskan FC Microplate Photometer (ThermoFisher Scientific) and incubated at 37 °C with background shaking. OD_600_ readings were measured every 30 minutes. Growth experiments were repeated twice and the data combined.

### Motility assays

Motility assays were performed according to a previously published protocol^53^. Cultures were prepared as described above and their optical densities were adjusted. Swimming plates were inoculated by touching a sterile tip into the prepared culture, stabbing into an appropriate agar plate and incubation at 30 °C for 24 hours. Swarming plates were inoculated from a swimming plate that had been incubated for 24 hours and incubated at 37 °C for 16 hours. Halo radii were measured in 3 biological replicates with 3 technical replicates per experiment. The composition of motility plates was as following: swimming motility plates had 1% (w/v) Tryptone, 0.5% (w/v) NaCl, 0.3% (w/v) Agar; swarming motility plates contained 1% (w/v) Tryptone, 0.5% (w/v) Glucose, 0.5% (w/v) Agar.

### Assessment of biofilm formation

Overnight cultures were subcultured to an OD_600_ of 0.6–0.8. Eight wells of a 96 well plate were inoculated with 100 µl of each bacterial strain. Plates were sealed and incubated at 30 °C for 72 hours. Non-adhered planktonic cells were carefully removed and wells washed once in distilled water. 125 µl of 1% (w/v) crystal violet was added carefully to each well and incubated at room temperature for 20 minutes. After removing non-bound dye by washing twice with distilled water, 300 µl of 100% ethanol was added to extract the dye. Crystal violet absorbance in extracts was measured at 590 nm.

### Analysis of bacterial cell length using phase microscopy

Bacteria from exponential growth phase (OD_600_ of 0.6–0.8) were centrifuged at 13,000 × g before fixation in 4% (w/v) paraformaldehyde for 24 hours. Phase contrast microscopy was performed using an Eclipse Ni-E microscope (Nikon) and the bacterial cell length measured at x 1000 magnification using NIS-Elements software. Bacteria were measured in 20 fields of view; any bacteria in which the poles were not visible/dubious were disregarded. The mean length, standard error and standard deviation were measured for each bacterial strain.

### Scanning electron microscopy (SEM)

Bacteria were grown as above and after washing in PBS fixed in 2.5% (v/v) glutaraldehyde in PBS for 24 hours. Fixative was removed by two washes in PBS. 50 μl of the sample was added to a glass coverslip treated with Poly-L-Lysine for 10 minutes. A secondary fixation was carried out in 1% (w/v) osmium tetroxide for 90 minutes. Samples were washed in deionised water several times followed by subsequent 30 minute washes in 30%, 50%, 70% and 90% (v/v) ethanol. Two final 30 minute washes in 100% analytical grade ethanol were performed. The slides were washed in 3:1 ethanol/hexamethyldisilazane (HMDS) for 30 minutes followed by 1:3 ethanol/HMDS for 30 minutes and finally 100% HMDS for 30 minutes (twice). Excess HDMS was removed and allowed to air dry overnight. Slides were mounted and sputter coated in gold (Emitech SC7640 sputter coater, 90 seconds, 20 mA Au). Bacteria were viewed on a Hitachi S3000H Scanning electron microscope with an accelerating voltage of 10 kV.

### Determination of minimum inhibitory concentrations

Overnight cultures were grown in Muller Hinton broth before subculturing (1:100) and growing for 3 hours at 37 °C. Cultures were diluted (1:10) and used to inoculate 96 well microtiter plates containing 2-fold serial dilutions of; ceftazidime, co-trimoxazole, doxycycline, meropenem, imipenem or carbenicillin (Sigma Aldrich) with a final inoculum of 10^5^ bacteria per well (confirmed by CFU counts). Plates were sealed and statically incubated for 24 hours at 37 °C before the MIC results were recorded. The MIC was the lowest concentration of antibiotic for which there was no visible bacterial growth. Experiments were repeated 3 times.

### Ethics statement

All investigations involving animals were carried out under a UK Home Office Project Licence, according to the requirements of the Animal (Scientific Procedures) Act 1986. Ethical approval was granted by our local (Defence Science and Technology Laboratory) ethical review process according to the requirements of the Animal (Scientific Procedures) Act 1986.

### BALB/c mouse infection

6–8-week old female BALB/c mice (Charles River) were randomised and caged in groups of 5 in a rigid walled isolator in an ACDP level 3 laboratory and allowed to acclimatise for at least 7 days. Challenge doses were prepared by inoculating 50 ml of LB with 50 µl of a frozen bacterial stock. Cultures were incubated at 37 °C with shaking at 180 rpm for 24 hours. The culture was adjusted to an OD_600_ of 0.35–0.38 (approximately 1 × 10^8^ CFU). 10-fold serial dilutions were prepared for use in the study. Mice were challenged with approximately 1 × 10^4^ CFU (confirmed by CFU counts) of wildtype or ∆*ltgG* via the intraperitoneal route (IP) route. 10 mice were used per group. The mice were observed for clinical signs of infection and mortality for 35 days. At day 35 all remaining survivors were culled and the spleen, liver and lungs were harvested. The organs were weighed and homogenised through a 40 µm cell strainer (FisherScientific) into 1 ml PBS and the bacterial load enumerated by CFU counts. CFU/g was calculated by dividing CFU/ml by weight of the organ. The remaining homogenate was added to 10 ml LB and incubated statically at 37 °C for 7 days before checking for *B*. *pseudomallei* growth by performing a streak onto LA.

### Cloning and expression of recombinant LtgG

The LtgG open reading frame (minus N’ terminal signal sequence) was PCR amplified from *B*. *pseudomallei* K96243 genomic DNA. The PCR product was cloned into pET15bTEV into *Nde*I and *Bam*HI restriction sites. *E*. *coli* C41 (DE3) was transformed with pET15bTEV-LtgG or pET15bTEV-LtgG D343A. Bacterial strains were grown to an OD_600_ of 0.6–0.8 and protein expression induced with 0.05 mM IPTG followed by incubation at 16 °C overnight. LtgG and LtgG D343A were purified using a Ni-NTA resin and size exclusion chromatography (SEC) on a 16/600 Superdex 200 pg column using ÄKTA™ Purifier system (GE Healthcare Life Sciences). SDS-PAGE and Western blots using primary anti-polyhistidine IgG antibodies (Sigma) and secondary alkaline-phosphatase conjugated anti-mouse antibodies (Sigma) were performed to assess purity of the protein. SIGMAFAST^TM^ BCIP/NBT substrate was used for detection of band according to the manufacturer instruction.

### LtgG crystallisation and structure determination

Purified recombinant LtgG, without the predicted N′ terminal signal sequence (356 residues), was concentrated to 5 mg/ml. LtgG crystals were grown in 0.1 M Bis-tris propane (pH 7.5), containing 0.2 M potassium sodium tartrate and 14% PEG 8K by mixing 1.5 µl drops of protein with an equivalent volume of crystallisation buffer using sitting-drops. Crystals were grown at 4 °C over 14 days, frozen in cryo-preservative – (crystallisation buffer containing 30% glycerol) and stored in liquid nitrogen prior to data collection. Diffraction data were collected at the Diamond Light Source and were processed with iMosflm. Phases were determined by molecular replacement with Phaser^54^ using the structures of LtgG homologues; MltA from *N*. *gonorrhea* (38% sequence identity over 396 residues; PDB: 2G5D) and *E*. *coli* (34% identity over 307 residues; PDB: 2GAE) as search models. Models were optimised using cycles of manual refinement with Coot and refinement in Refmac5^55^, part of the CCP4 software suite^56^, and in Phenix^57^.

### Assessment of muralytic activity

Proteins were run on an SDS-PAGE gel containing 1.5% (w/v) lyophilised *M*. *luteus*. Gels were washed twice in distilled water (10 minutes per wash), before incubation at 37 °C for 18 hours in refolding buffer (25 mM Tris pH 7 containing 1 mM MnCl_2_). Clearance bands were enhanced by staining in 0.1% (w/v) methylene blue containing 0.01% (w/v) potassium hydroxide.

A turbidometry assay was performed as described previously^14^. Briefly, lyophilised *M*. *luteus* was resuspended in 2 mM citric acid pH 6 and 200 µl dispensed into wells of a 96 well plate. Triplicate wells were inoculated with recombinant LtgG, heat inactivated LtgG, a lysozyme positive control (all at a concentration of 20 µg/ml) or buffer only controls. OD_600_ readings were measured using a Varioskan plate reader (ThermoFisher Scientific) every 10 minutes. The experiment was repeated three times and the data combined. Finally, peptidoglycan was purified from *E*. *coli* as described earlier^58^ and labelled with FITC^59^. For activity assays, 5 µg of LtgG was mixed with 50 µg of labelled peptidoglycan in buffer containing 25 mM TrisCl, pH 8.0 and incubated at 30 or 37 °C for 18 hours before the reaction was stopped. Samples were passed through a 0.22 µm before measuring fluorescence at 525 nm.

### Statistical analysis

Statistical analysis was performed using GraphPad Prism v 6.02. Growth, motility and biofilm data were combined and analysed using a one-way ANOVA, in which wildtype data was compared to each set of mutant data. Animal survival data were compared using the Mantel-Cox-Log-Rank test. A *p*-value of <0.05 was considered significant.

## Supplementary information

Supplementary File

## Data Availability

Structure of LtgG protein has been deposed in the PDB https://www.wwpdb.org; accession numbers PDB code is 6QK4.
