# Supplementary material for: The lytic transglycosylase, LtgG, controls cell morphology and virulence in Burkholderia pseudomallei
Source: Sci Rep. 2019 Jul 30;9:11060. doi: 10.1038/s41598-019-47483-z (PMC6667503; doi:10.1038/s41598-019-47483-z)
Supplement: Supplementary file 1 — Supplementary File [file 41598_2019_47483_MOESM1_ESM.pdf]

Supplementary Information

The lytic transglycosylase, LtgG, controls cell morphology and virulence in *Burkholderia pseudomallei*.

Christopher H. Jenkins[1,2], Russell Wallis[1,3], Natalie Allcock[4], Kay B. Barnes[2], Mark I. Richards[2], Joss M. Auty[5], Edouard E. Galyov[1,6], Sarah V. Harding[2,5], Galina V. Mukamolova[5*]

[1]Department of Infection, Immunity and Inflammation, University of Leicester, Leicester, UK; [2]Defence Science and Technology Laboratory, Chemical, Biological and Radiological Division, Porton Down, Salisbury, Wiltshire, UK; [3]The Leicester Institute of Structural and Chemical Biology, Henry Wellcome Building, University of Leicester, Leicester, UK; [4]Electron Microscopy Facility, Core Biotechnology Services, University of Leicester, Leicester, UK; [5]Department of Respiratory Sciences, University of Leicester, Leicester, UK; [6]Department of Genetics and Genome Biology, University of Leicester, Leicester, UK

*To whom correspondence should be addressed: Christopher H. Jenkins (cjenkins@dstl.gov.uk) and Galina V. Mukamolova, Department of Respiratory Sciences, College of Life Sciences, University of Leicester, Maurice Shock building, University Road, Leicester, LE1 9HN, UK; gvm4@leicester.ac.uk

---

Supplementary Figures 1-4

Supplementary Tables 1-5

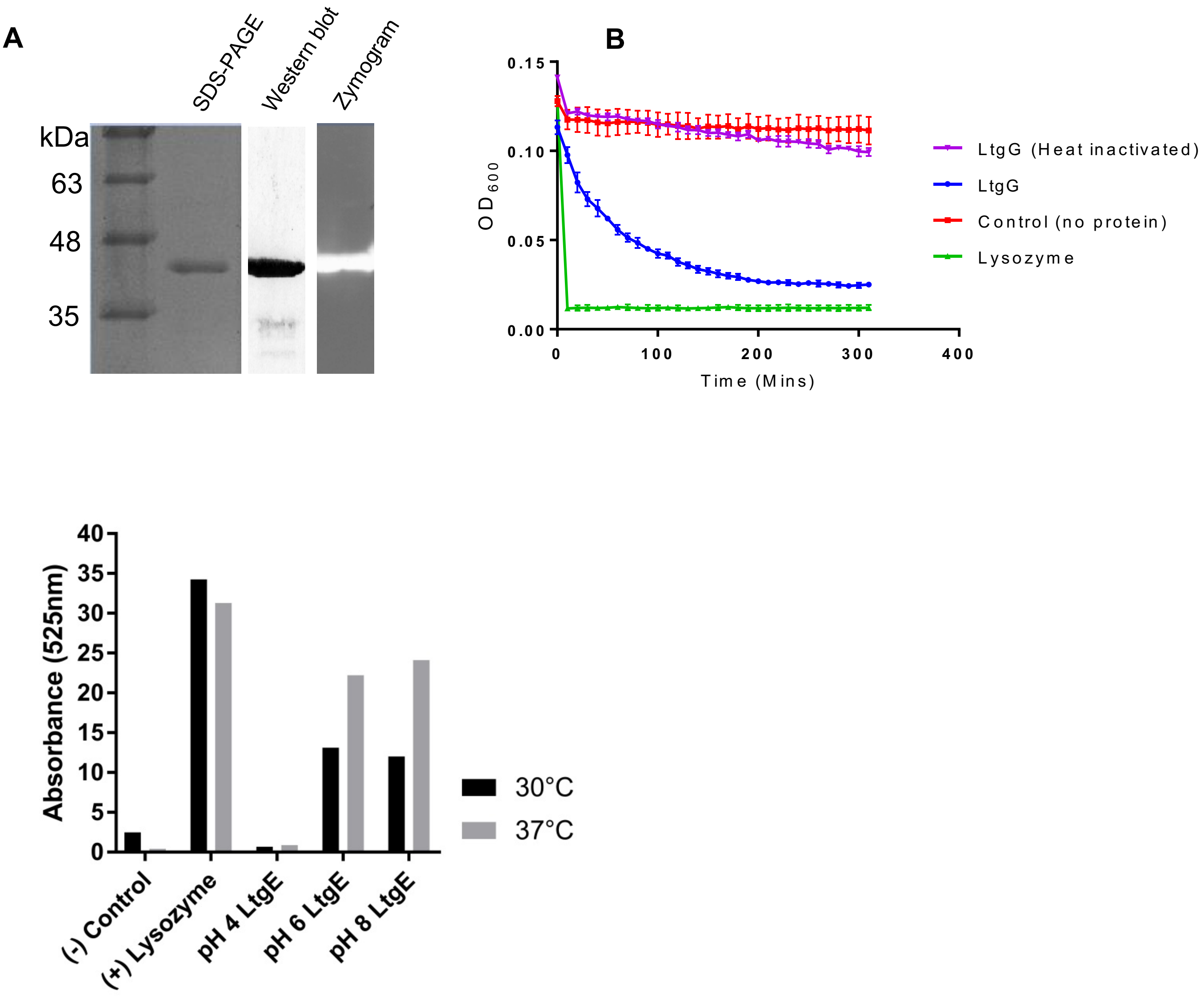

Supplementary Figure 1. Recombinant LtgG possesses muralytic activity. (A) His-tagged recombinant *B. pseudomallei* LtgG was purified from *E. coli*, visualised by Western blot and used in a zymogram, producing a clearance band. (B) Turbidometry assay was performed as described in the methods. OD_{600} readings were measured every 10 minutes with activity seen by a reduction in optical density. The experiment was performed in triplicate. (C) Digestion of FITC labeled *E. coli* peptidoglycan by recombinant LtgG. Results of representative experiment are shown.

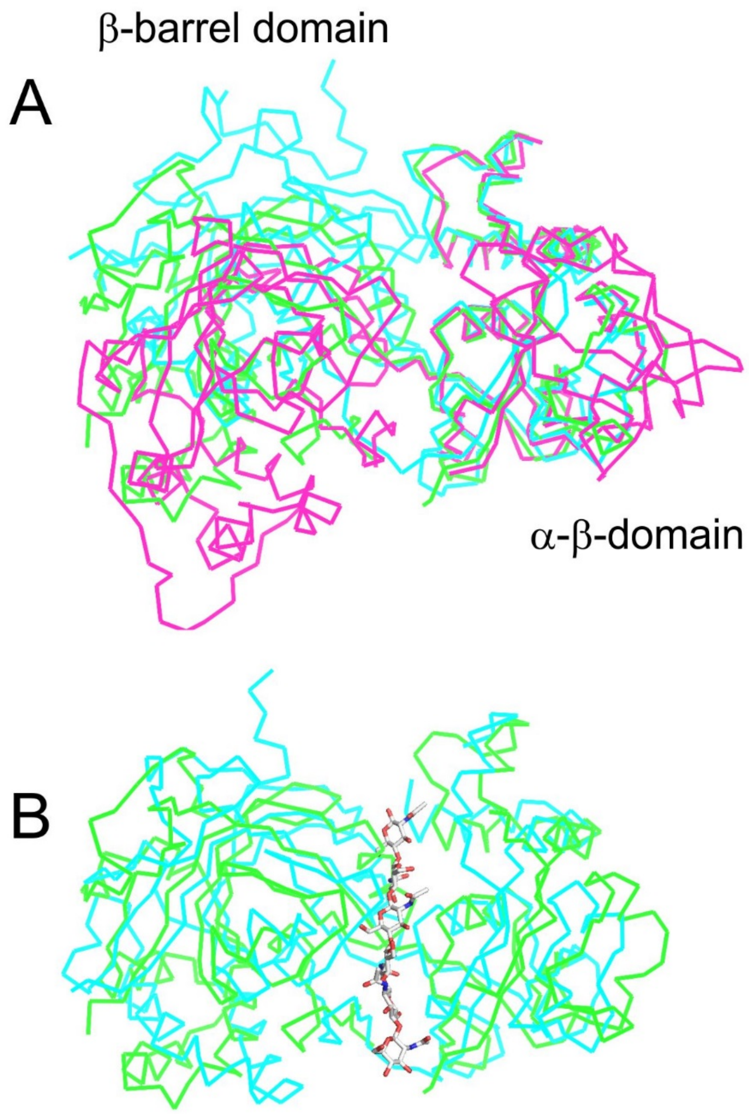

Supplementary Figure 2. Comparison of the structure of LtgG with MltA of *E. coli* and *N. gonorrhoeae*. (A) Structures of *E. coli* (PDB: 2GAE; cyan) and *N. gonorrhoeae* MltA (2G6G; magenta) aligned with LtgG (green) using the α-/β-domain only. The β-barrel domain is rotated by ~40º and ~20º, respectively. (B) Overlay of LtgG with *E. coli* MltA bound to a chitohexaose substrate (PDB: 2PI8).

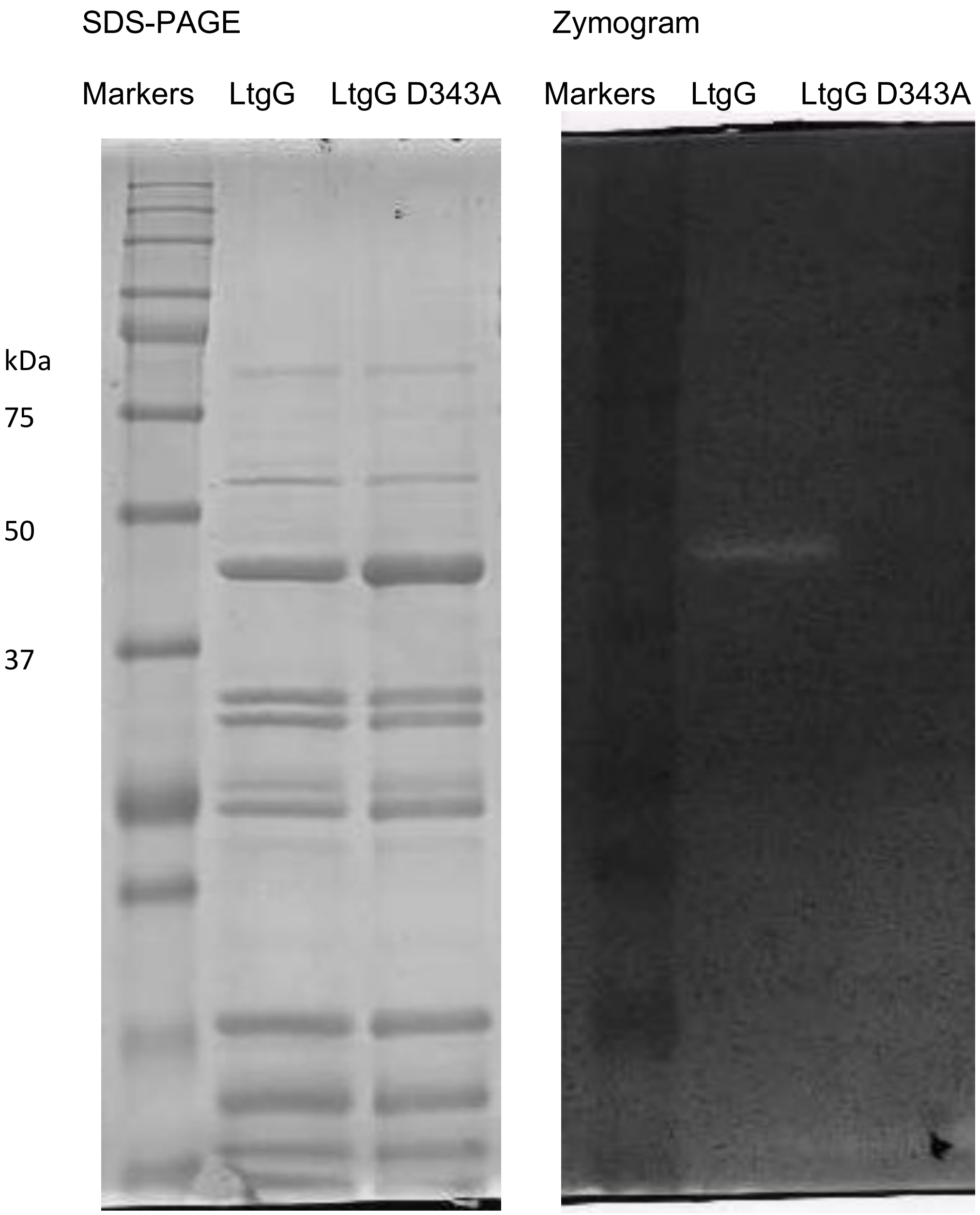

Supplementary Figure 3. Assessment of muralytic acivity of LtgG and its mutant LtgG D343A (uncropped images).

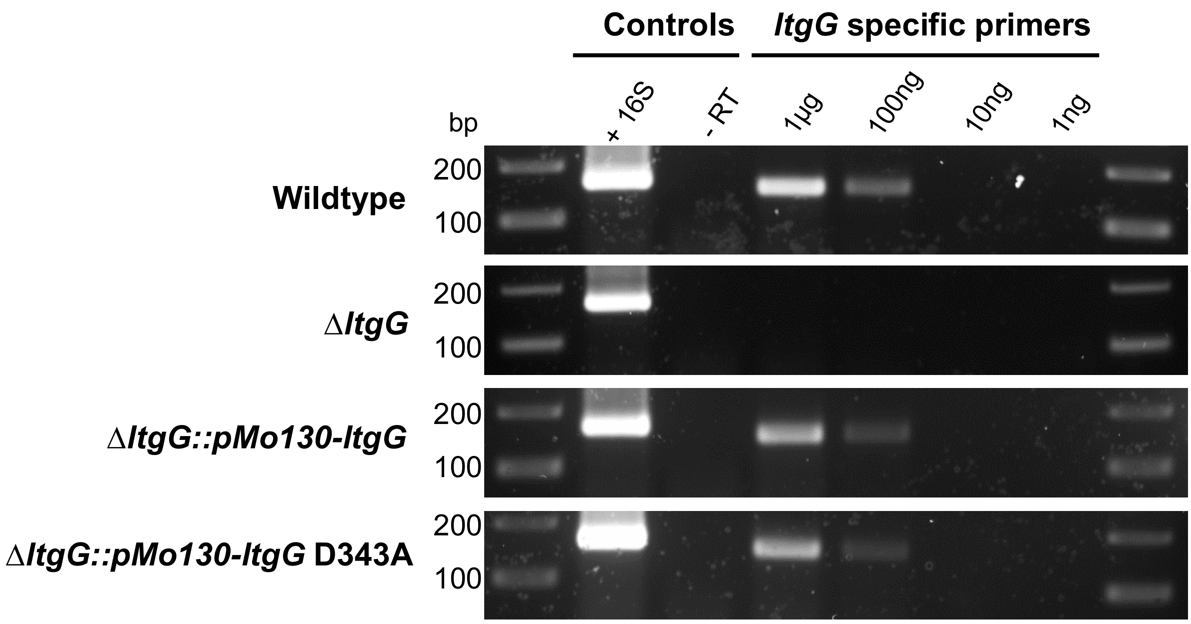

Supplementary Figure 4. Reintroduction of *ltgG* into the Δ*ltgG* mutants restores LtgG expression to the wildtype level. RNA was isolated from a growing bacterial culture and cDNA synthesised as described in the methods section. Serial dilutions of cDNA were used for PCR with *ltgG* specific primers. Amplification of the 16S ribosomal RNA gene was used as a positive control (+16S rRNA) which shows RNA presence in production of cDNA. A reverse transcriptase control (-RT) shows no contamination with gDNA.

Table S1. Lytic transglycosylases identified in *B. pseudomallei* K96243

Gene accession number	Protein designation used in this study	Homologues in other bacteria	Family	UniProtKB	Length (amino acids)	Molecular weight (kDa)	Predicted catalytic residue(s)	LTG domain (PFAM)
BPSL0006	LtgA	Slt	1A	Q63Z31	161	18.1	E48	Slt_1 (PF01464)
BPSL0262	LtgB	MltE1	1G	Q63YC6	651	72.4	E512	Slt_1 (PF01464)
BPSL1345	LtgC	MltD	1D	Q63V96	553	60.6	E154	Slt_1 (PF01464)
BPSL2506	LtgD	MltB	3A	Q63S16	450	48.9	D357	Slt_2 (PF13406)
BPSL1435	LtgE	MltG	5A	Q63V09	339	36.5	E223	YceG (PF02618)
BPSL2630	LtgF	MltE2	1G	Q63RP2	355	36.6	E182	Slt_1 (PF01464)
BPSL3046	LtgG	MltA	2A	Q63QH7	372	39.9	D300, D331, D343	MltA (PF03562)
BPSL3276	LtgH	RlpA	6A	Q63PU6	211	22.3	D156?	DPBB_1 (PF03330)

Table S2. Bacterial strains used and generated in the

	Strain	Properties	Reference/Source
E. coli	C41(DE3)		Lucigen
	S17-1(λ*pir*)	S17-1 containing a λ prophage carrying the *pir* gene	(1)
B. pseudomallei	K96243	Wildtype strain	(2)
	Δ*ltgC*	Unmarked deletion in BPSL1345	This study
	Δ*ltgD*	Unmarked deletion in BPSL2506	This study
	Δ*ltgF*	Unmarked deletion in BPSL2630	This study
	Δ*ltgG*	Unmarked deletion in BPSL3046	This study
	Δ*ltgGC*	Unmarked deletions in BPSL1345, BPSL3046	This study
	Δ*ltgGCF*	Unmarked deletions in BPSL1345, BPSL2506, BPSL3046	This study
	Δ*ltgCFD*	Unmarked deletions in BPSL1345, BPSL2506, BPSL2630	This study
	Δ*ltgGCFD*	Unmarked deletions in BPSL1345, BPSL2506, BPSL2630, BPSL3046	This study
	Δ*ltgG*::pMo130-*ltgG*	Unmarked deletion in BPSL3046 complemented *in cis* with BPSL3046	This study
	Δ*ltgGCFD*::pMo130-*ltgG*	Unmarked deletions in BPSL1345, BPSL2506, BPSL2630, BPSL3046 complemented *in cis* with BPSL3046	This study
	Δ*ltgGCFD*::pMo130-*ltgG* D343A	Unmarked deletions in BPSL1345, BPSL2506, BPSL2630, BPSL3046 complemented *in cis* with BPSL3046 containing a D343A mutation	This study

Table S3. Growth parameters of *B. pseudomallei* strains

Strain	Maximum growth rate (h^{-1})	Doubling time, h	OD_{600} at 24 h
Wildtype	0.619±0.054	1.13±0.11	0.89±0.03
Δ*ltgF*	0.637±0.020	1.09±0.03	0.96±0.01
Δ*ltgF*	0.604±0.025	1.15±0.05	0.85±0.07
Δ*ltgF*	0.581±0.030	1.20±0.06	0.84±0.05
Δ*ltgG*	0.591±0.026	1.18±0.05	0.93±0.03
Δ*ltgGCFD**	0.558±0.022	1.24±0.05	0.056±0.07
Δ*ltgGCFD*:: pMo130-*ltgG*	0.602±0.023	1.15±0.04	0.89±0.01

Growth rate μ and doubling time t_d were calculated using the following formulae: μ=2.303(lgOD4.5-LgOD0.5)/4, where OD4.5 is optical density at 4.5 hour and OD0.5 is optical density at 0.5 hour; t_d=Ln2/μ
Average values with STDV (N=7) shown.
*Growth rate, doubling time and OD at 24 h for Δ*ltgGCFD* were significantly statistically different from those in wildtype and Δ*ltgGCFD*::pMo130-*ltgG* (P<0.05, one way ANOVA).

Table S4. Data collection and refinement statistics.

PDB code	To be updated on submission
Data collection	
Beam line	Diamond I03
Space group	P 3_2 21
a, b, c, Å	59.6 59.6 168.3
α, β, γ, º	90, 90, 120
Resolution, Å	56.1 – 1.73 (1.78 – 1.73)
$R_{sym, \%}$	11.5 (70.2)
CC(1/2)	1.0 (0.80)
$I/\delta I$	16.1 (2.6)
Completeness	100 (100)
Redundancy	16.3 (8.6)
Refinement	
Resolution, Å	51.6 - 1.73 (1.79 - 1.73)
No. reflections	37097 (3640)
R_{work}/R_{free}	0.217/0.249
No. atoms	2833
Protein	2536
Water	297
B-factors, $Å^2$	34.6
Protein	34.1
Water	38.9
Bond lengths, Å	0.004
Bond angles, º	0.91

The highest resolution shell is shown in parenthesis.

Table S5. Primers used in the study.

Name	Sequence (5' – 3')	Purpose
16S_For	GACACGGCCCAGACTCCTAC	16S for RT-PCR (control)
16S_Rev	CCGGTACCGTCATCCACTCC	16S for RT-PCR (control)
LtgC_Screen_For	CGTTCACGTTGATGCGCGAGT	Confirmation screening of Δ*LtgC*
LtgC_Screen_Rev	ATCACGATCACCGGTAAGACG	Confirmation screening of Δ*LtgC*
LtgC_Up_For (*Hin*dIII)	ATAAAGCTTACAACTACATCTGGCTCGTGT	Upstream flanking region for *LtgC* deletion mutant
LtgC_Up_Rev (*Bam*HI)	ATAGGATCCTTGCGCAAGTAGTCCGAA	Upstream flanking region for *LtgC* deletion mutant
LtgC_Down_For (*Bam*HI)	ATAGGATCCAAGTAAGACGAAAGGGCGCGC	Downstream flanking region for *LtgC* deletion mutant
LtgC_Down_Rev (*Xba*I)	ATATCTAGAAACCCGCAGAACGCATACAGC	Downstream flanking region for *LtgC* deletion mutant
LtgD_Screen_For	TGTAATCGATGTGCGCCTGCG	Confirmation screening of Δ*LtgD*
LtgD_Screen_Rev	AGGTTCGACTGCTCGCTTGCG	Confirmation screening of Δ*LtgD*
LtgD_Up_For (*Hin*dIII)	ATAAAGCTTTCGTAGTCGGCTTCGACGAGC	Upstream flanking region for *LtgD* deletion mutant
LtgD_Up_Rev (*Bam*HI)	ATAGGATCCATTGCGGCTGCGCCGGCTCTT	Upstream flanking region for *LtgD* deletion mutant
LtgD_Down_For (*Bam*HI)	ATAGGATCCGAATGACGCCGACGCGGCGCG	Downstream flanking region for *LtgD* deletion mutant
LtgD_Down_Rev (*Xba*I)	ATATCTAGAAAGACACGATCGGCAATA	Downstream flanking region for *LtgD* deletion mutant
LtgF_Screen_For	ACGGTACATATACCGTTTCTC	Confirmation screening of Δ*ltgF*
LtgF_Screen_Rev	TCAATATCCGCGTCGGAAACA	Confirmation screening of Δ*ltgF*
LtgF_Up_For (*Hin*dIII)	ATAAAGCTTATCTTTGTATTTCATGAAGAA	Upstream flanking region for *ltgF* deletion mutant
LtgF_Up_Rev (*Xho*I)	ATACTCGAGCAACATCTGCGCATGCCGCTCACT	Upstream flanking region for *ltgF* deletion mutant
LtgF_Down_For (*Xho*I)	ATACTCGAGACCTGACGAAATCGGCGGCAA	Downstream flanking region for *ltgF* deletion mutant
LtgF_Down_Rev (*Xba*I)	ATATCTAGAGCAAGCTGTTCGGGCGCTAAC	Downstream flanking region for *ltgF* deletion mutant
LtgG_Comp130_For (*Bgl*II)	ATCGAGCCAGATCTACGCTGCACTGAGCGCGGCGTCGC	*ltgG* including upstream region for insertion into pMo130 for *in cis* complementation

LtgG_Comp130_Rev (*EcoRV/Sma*I)	GGATATCCCCGGGTCACGAATTCGGAAACAGCAGCCA	*ltgG* including upstream region for insertion into pMo130 for *in cis* complementation
LtgG_compUp_For (*Nhe*I)	TGCGATCGCTAGCCATCAAACGGCCGACCGTGGTGGACATTC	Upstream flanking region for integration of in cis complementing plasmid downstream of *bpsl3330*
LtgG_compUp_Rev (*Bgl*II)	CAGCTTTCAGATCTGGCTCGATCAAAATCCCTTCCAGTAATCG	Upstream flanking region for integration of in cis complementing plasmid downstream of *bpsl3330*
LtgG_GS_For	TATCAGTACGCGCTCTACC	*ltgG* gene specific
LtgG_GS_Rev	CTGCACCTGCAGAAAGAA	*ltgG* gene specific
LtgG_Asp343Ala_Rev	GCCGAGCCCCAGAAATAGGCGGCCCGCACGCCGCCCTTG	Insertion of point mutation (Asp343Ala) in LtgG
LtgG_Asp343Ala_For	CAAGGGCGGCGTGCGGGCCGCCTATTTCTGGGGGCTCGGC	Insertion of point mutation (Asp343Ala) in LtgG
LtgG_Screen_For	GCCGCGCACGCTGCACTGAGCGCG	Confirmation screening of Δ*ltgG*
LtgG_Screen_Rev	CGATACGTCCGGTCGCGCGTCGAA	Confirmation screening of Δ*ltgG*
LtgG_Up_For (*Hin*dIII)	ATAAAGCTTAGCTATTTGCCGGAACAATCC	Upstream flanking region for *ltgG* deletion mutant
LtgG_Up_Rev (*Xho*I)	ATACTCGAGGCTAAAACCCATACAATGTCC	Upstream flanking region for *ltgG* deletion mutant
LtgG_Down_For (*Xho*I)	ATACTCGAGAATTCGTGATCCTGTGATCTC	Downstream flanking region for *ltgG* deletion mutant
LtgG_Down_Rev (*Xba*I)	ATATCTAGACGCGCATCCACGCTTCGTCAG	Downstream flanking region for *ltgG* deletion mutant
LtgG_pET15_For (*Nde*I)	ACACATATGTCTGTCGGCTCGCCGGTGCGGCAG	Non-secreted LtgG
LtgG_pET15_Rev (*Bam*HI)	ACTGGATCCTCACGAATTCGGAAACAGCAGCCA	Non-secreted LtgG
